# SUPPORTING OLDER ADULTS WHO LIVE ALONE: UNDERSTANDING THEIR UNMET NEEDS USING NHATS SURVEY DATA

**DOI:** 10.1093/geroni/igad104.2251

**Published:** 2023-12-21

**Authors:** Marissa Bergh, Aasha Raval, Jasmine Travers

**Affiliations:** New York University, New York City, New York, United States; NYU School of Global Public Health, New York City, New York, United States; New York University, New York City, New York, United States

## Abstract

Research suggests that older adults living alone in the community are at higher risk for poor health-related outcomes due to lack of cohabitants to support their health needs. Using National Health and Aging Trends Study (NHATS) data, we identified community-residing older adults (≥65 years) with assistance needs for Instrumental Activities of Daily Living (IADLs), Activities of Daily Living (ADLs), or mobility. We used bivariate analyses to compare the unmet needs related to IADL, ADL, and mobility tasks between those living alone and those living with others. Of the 3,458 individuals identified, 28.9% (n=1001) lived alone. Those living alone were more likely to be women, unpartnered, and lacking informal caregivers. There was no difference in perceived overall health scores between the groups. Individuals living alone experienced more mobility and IADL-related unmet needs. In the month prior to survey completion, those living alone were more likely to forgo getting out of bed (p=0.031), grocery shopping (p< 0.001), cooking (p< 0.001), and paying bills (p=0.025) due to lack of available assistance. Our findings suggest that older adults living alone may need higher levels of formal assistance with IADLs to remain living in the community. Given the key role IADLs play in promoting quality of life and a person’s ability to remain independent and engaged in the community, it is important to consider IADLs in addition to ADLs when assessing formal care needs for those living alone. Policy makers should prioritize the specific needs of those living alone when developing national aging strategies.

